# Validity of the Montreal Cognitive Assessment and the HIV Dementia Scale in the assessment of cognitive impairment in HIV-1 infected patients

**DOI:** 10.1007/s13365-015-0324-4

**Published:** 2015-02-13

**Authors:** M. A. M. Janssen, M. Bosch, P. P. Koopmans, R. P. C. Kessels

**Affiliations:** 1 Department of Medical Psychology, Radboud University Nijmegen Medical Center, P.O. Box 9101, 6500 HB Nijmegen, The Netherlands; 2 Department of Internal Medicine, Radboud University Nijmegen Medical Center, P.O. Box 9101, 6500 HB Nijmegen, The Netherlands; 3 Donders Institute for Brain, Cognition and Behaviour, Radboud University Nijmegen, P.O. Box 9010, 6500 GL Nijmegen, The Netherlands

**Keywords:** HIV/AIDS, Cognitive impairment, Screening tool, MoCA, HDS

## Abstract

The gold standard for evaluating cognitive impairments in HIV-infected patients is to administer an extensive neuropsychological assessment. This may, however, be time-consuming and hence not always feasible in the clinic. Therefore, several brief screening tools have been developed. This study determined the validity of the Montreal Cognitive Assessment (MoCA) and the HIV Dementia Scale (HDS) in detecting cognitive impairment using both the Frascati and cognitive impairment, no dementia (CIND) criteria to classify cognitive impairment in HIV-1 infected patients. The MoCA, HDS, and an extensive neuropsychological assessment, covering nine cognitive domains, were administered in a group of 102 HIV-infected patients who were all on cART and virologically suppressed for at least 1 year. Results show that the areas under the curve (AUCs) for both the MoCA and the HDS were statistically significant, using both the Frascati and the CIND criteria as gold standard. However, the AUCs for the MoCA and HDS did not differ significantly, regardless of the used classification criteria (Frascati: *z* = 0.37, *p* = 0.35; CIND: *z* = −0.62, *p* = 0.27). Sensitivity of both the MoCA and HDS were low for the recommended cutoff scores (Frascati: MoCA (<26) = 0.56, HDS (<11) = 0.26; CIND: MoCA (<26) = 0.55, HDS (<11) = 0.36). Cutoff scores with good sensitivity and adequate specificity could not be determined for both screening instruments. Therefore, the HDS and MoCA are not recommended as sole instruments to diagnose HIV-associated cognitive impairment.

## Introduction

The incidence of severe HIV-associated cognitive impairment has significantly declined since the introduction of combination antiretroviral therapy (cART), while milder forms of cognitive decrements continue to be prevalent and increase with age. Recent results from the CHARTER study demonstrated that 44 % of HIV-infected patients on cART, without marked comorbidities, fulfilled the criteria for milder forms of HIV-associated neurocognitive disorder (HAND) (Heaton et al. 2010). These neurocognitive impairments may have a great impact on functioning in vocational settings or on academic achievements. Furthermore, cognitively impaired patients are at greater risk of poor medication adherence (Hinkin et al. 2004).

The gold standard for adequately evaluating cognitive impairments is to administer an extensive neuropsychological assessment, as this method has a high sensitivity and specificity. A neuropsychological assessment typically covers a range of cognitive domains, such as memory, executive functioning, and attention, and can be used to classify levels of cognitive dysfunction in HIV using the widely used revised HAND criteria, referred to as the Frascati criteria (Heaton et al. 2010; Antinori et al. 2007). Patients are classified as asymptomatic or mildly impaired when they show a performance that lies 1 standard deviation (SD) below the demographically adjusted normative mean, yet within 2 SD in at least two cognitive domains. When a patient obtains a score of at least 2 SDs below the mean within at least two cognitive domains, he or she is classified as severely impaired. Gisslén et al. (2011) pointed out, however, that a cutoff of −1 SD to classify impairment may lead to overestimation of the real prevalence of cognitive impairment. That is, given the normal distribution, 15.9 % of any population will perform worse than 1 SD below the normative mean on a given test. Another way to determine cognitive impairment is to use the criteria “cognitive impairment, no dementia” (CIND). Patients are classified as having CIND when one or more of the cognitive domains are impaired, which may result in less false positives (Van den Berg et al. 2005). The CIND criteria define impairment as an average performance >1 on the tasks in a cognitive domain (a score of 0 is obtained when the performance is in the normal range (−1 SD to 1 SD), a score of 1 when the performance is below average (−1 SD to −1.65 SD) and a score of 2 when the performance is impaired (less than −1.65 SD). The CIND criteria are somewhat more stringent because they require at least one of the test scores in a cognitive domain to be lower than −1 SD to be classified as impaired.

The problem with extensive neuropsychological testing, however, is that it is often time-consuming and requires trained personnel to administer, score, and interpret (Koski et al. 2011; Overton et al. 2013). To overcome this limitation, several brief screening tools to detect cognitive impairment in HIV-infected patients have been developed over the years (Valcour et al. 2011). An example of such a short screening test is the HIV Dementia Scale (HDS) (Power et al. 1995). This test consists of items addressing memory, attention, psychomotor functioning, and visuoconstruction. The HDS has been shown to be an adequate screening tool in HIV-infected patients in detecting severe cognitive impairment, but has been shown to be insensitive to milder forms of cognitive deficits (Valcour et al. 2011; Bottigi et al. 2007; Carey et al. 2004; Zipursky et al. 2013).

Another widely used screening method to detect cognitive impairment is the Montreal Cognitive Assessment (MoCA) (Nasreddine et al. 2005). This screening test takes approximately 10–15 min to administer and consists of 30 items measuring eight cognitive domains. The MoCA is sensitive in differentiating milder forms of cognitive impairments and has been validated in patients with Parkinson’s disease, Huntington’s disease, substance abuse, and mild cognitive impairment (MCI) (Freitas et al. 2012; Larner 2012; Videnovic et al. 2010; Zadikoff et al. 2008; Wester et al. 2013; Thissen et al. 2010). However, to date, few studies have investigated the validity of the MoCA in HIV-infected patients (Koski et al. 2011; Overton et al. 2013; Hasbun et al. 2012; Milanini et al. 2014). Most studies used the Frascati criteria to classify cognitive impairment and none of these studies have directly compared the widely known HDS to the MoCA. Ours is the first study that investigates the validity of the MoCA and the HDS in HIV-infected patients in relation to two sorts of clinical criteria to determine cognitive impairment.

The current study focused on a cohort of HIV-infected patients who were all on cART and virologically suppressed for at least 1 year. The primary objective of this study was to determine the validity of the MoCA and the HDS in detecting cognitive impairment as measured with an extensive neuropsychological test battery, and impairment classified with both the Frascati criteria and the CIND criteria. Sensitivity, specificity, and areas under the curve (AUCs) of the HDS and the MoCA were assessed. Furthermore, we aimed to determine sensitive and specific cutoff scores for both the MoCA and the HDS.

## Methods

### Participants

A total of 102 HIV-1 infected patients were included between January 16, 2012 and January 31, 2014. Consecutive patients were recruited through their treating physicians via the outpatient clinic for infectious diseases at the Department of Internal Medicine in the Radboud University Medical Center in Nijmegen and at the same department in the Rijnstate Hospital Arnhem. Patients were eligible if they were between 18 and 70 years old, fluent speaker of the Dutch language, had no current drug or alcohol addiction, and no history of psychiatric or neurological disorder (unrelated to HIV-1 infection in the patients). Inclusion criteria were an HIV-1 infection and absence of active opportunistic infections, pregnancy, malignancy, and neurosyphilis. HIV status of all patients was determined by enzyme-linked immunosorbent assays (ELISA) and a Western blot confirmatory test. Patients were selected regardless of the presence of signs or symptoms of suspected cognitive impairment or subjective cognitive complaints. Medical ethical approval was obtained for this study, and written informed consent was obtained from all participants.

### Neuropsychological assessment

Participants completed an extensive neuropsychological test battery measuring nine major cognitive domains. Tests that are sensitive to measure small or moderate differences in ability were chosen and were administered by trained neuropsychologists. The allocation of tests to the domains was made a priori, according to standard neuropsychological practice, psychometric properties of the tests, and cognitive theory (Lezak et al. 2012). Also, the domains and tests were based on previous studies that examined cognitive impairment in HIV-infected patients (Heaton et al. 2010; Janssen et al. 2013).

*Abstract reasoning* was assessed by the Raven Advanced Progressive Matrices (12-item short form) (Raven et al. 1993). *Language* was assessed with a letter fluency tasks (“K-O-M”; 1 min per letter) (Schmand et al. 2008). The domain *Speed of information processing* included the Digit-Symbol Substitution subtest from the WAIS-III, the Trail Making Test part A (TMT-A), and the Stroop Color-Word Test (cards I and II) (Wechsler 1955; Reitan 1958; Rey 1964; Stroop 1935). *Learning* was assessed both verbally and nonverbally with the Dutch version of the Rey Auditory Verbal Learning Test (RAVLT, immediate memory: total score on trials 1–5) and the Location Learning Test–Revised (LLT-R, immediate memory: total score on trials 1–5), respectively (Rey 1964; Bucks et al. 2011). The domain *Memory* was also assessed verbally and nonverbally with the same tasks as the domain *Learning* with the delayed recall trial of both tasks. *Executive function* consisted of three subdomains: *Concept shifting*, *Planning*, and *Response inhibition. Shifting* was measured with the Brixton Spatial Anticipation Test and with the interference score of the Trail Making Test part B (TMT-B) (Burgess and Shallice 1997; Reitan 1958). *Planning* was assessed with the Zoo Map test from the Behavioural Assessment of the Dysexecutive Syndrome (BADS) (Wilson et al. 2003). *Response inhibition* was assessed by the Stroop Color-Word Test (Stroop 1935). Here, the Stroop interference score was computed, using the following formula: (time needed for card III − time needed for card II) / time needed for card II) (Stuss et al. 2001). *Attention/Working memory* was measured with the 2.0 and 1.6 interstimulus interval (ISI) trials of the Paced Auditory Serial Addition Test (PASAT) (60 items per trial), the Letter-Number Sequencing subtest from the Wechsler Adult Intelligence Scale, third edition (WAIS-III), and the Corsi Block Tapping task (Aarnoudse et al. 1995; Wechsler 1955; Kessels et al. 2000). The domain *Motor function* was measured with the Grooved Pegboard Test (administered for the dominant and nondominant hand) (Heaton et al. 1992). Finally, *Visuoconstruction* was measured with the copy trial of the Rey-Osterrieth Complex Figure Test (Rey 1941).

Symptom validity was measured with the short version of the Amsterdam Short-Term Memory Test (ASTM) (cutoff score <42) (Schmand et al. 1999). To assess whether patients had subjective cognitive complaints, all participants completed the Cognitive Failures Questionnaire (CFQ; Broadbent et al. 1982), using a cutoff score of 1.65 SD above the age-adjusted normative mean for the CFQ total score (Ponds et al. 2006). Education level was recorded using seven categories in agreement with the Dutch educational system (1 = less than primary school; 7 = academic degree). These levels match closely to the following categories in year of education as used in the Anglo-Saxon world (Bouma et al. 2012; Oosterman et al. 2014): level 1, incomplete primary education 1–5 years; level 2, primary education 6 years; level 3, incomplete lower secondary education 7–8 years; level 4, lower general secondary education 7–9 years; level 5, vocational education 7–10 years; level 6, higher general secondary/higher vocational/pre-university education 7–16 years; and level 7, academic degree 17–20 years. Premorbid intellectual level (estimated IQ) was estimated with the Dutch version of the National Adult Reading Test (Schmand et al. 1992).

### Montreal Cognitive Assessment

Each participant completed the Dutch version of the MoCA (Nasreddine et al. 2005). The MoCA consists of 13 tasks measuring the following eight cognitive domains: visuospatial/executive, naming, memory, attention, language, abstraction, delayed recall, and orientation. The MoCA takes approximately 10–15 min to complete. A total score was calculated by summing scores of the 13 tasks. The maximum score possible is 30 points, with a cutoff score of ≤26 indicative of cognitive impairment. One point was added for each participant with 12 or fewer years of formal education.

### HIV Dementia Scale

Each participant also completed the HDS (Power et al. 1995). This test consists of four items measuring four cognitive domains including memory, attention, motor speed, and visuoconstruction. A total score was calculated by summing scores of the four items, with a maximum score of 16. A score of <11 gives an indication of cognitive impairment.

### Data analysis

Neuropsychological impairments were classified per task using age- and education-adjusted normative data (i.e., using 1 SD and 1.65 SD below the normative mean as cutoff scores for mild and severe impairment, respectively) (Bouma et al. 2012; Lezak et al. 2012; Van den Berg et al. 2005). The performance on the neuropsychological assessment as a whole was classified as either “impaired” or “unimpaired” for each patient using both the Frascati and CIND criteria. In the Frascati criteria, impairment of 1 SD below the normative mean must to be present in at least two domains for a participant to be classified as “cognitively impaired.” Furthermore, at least one of the ability deficits has to be outside the motor and sensory perceptual domain, in agreement with the updated nosology for HIV-associated neurocognitive disorders by Antinori et al. (2007). Performance on the CFQ was used to classify cognitively impaired patients in accordance with the Frascati criteria as having asymptomatic neurocognitive impairment (ANI; i.e., no subjective complaints, yet mild impairments on cognitive testing) or mild neurocognitive disorder (MND; i.e., both subjective complaints and mild impairments on cognitive testing). None of the patient fulfilled the criteria for HIV-associated dementia (HAD; i.e., all patients functioned independently at home).

Using the CIND criteria, a patient’s performance was classified as cognitively impaired if impairments were present in one or more of the individual cognitive domains. Performance on each test was rated as within the normal range (0), below average (1), or impaired (2). A score between −1 SD and 1 SD was defined as normal performance, a score between −1 SD and −1.65 SD as mildly impaired, and a score below −1.65 SD as impaired. A cognitive domain was classified as impaired when the average rating of tests in that domain was >1 (Van den berg et al. 2005).

In agreement with Woods et al. (2003), a performance below the cutoff of a symptom validity test is likely not the result of actual HIV-associated cognitive impairment and must therefore be regarded as an indication of underperformance due to suboptimal effort. Participants performing below this cutoff were removed from the statistical analyses.

Data were analyzed by using IBM SPSS version 19.0. Receiver operating characteristic (ROC) analyses were performed with the MoCA and the HDS as continuous variables and cognitive impairment, classified with both the Frascati and the CIND criteria, as state variable. The AUC was determined for each ROC curve, and cutoff scores for both screening tools were determined that had good sensitivity accompanied with an acceptable specificity. A cutoff score was defined as adequate if a sensitivity of >0.8 was accompanied by an acceptably low false-positive rate (specificity >0.6) (Blake et al. 2002).

## Results

Three patients performed below the cutoff of the symptom validity test and were removed from the analyses. Furthermore, two patients dropped out due to medical reasons unrelated to HIV status (recent CVA and severe epilepsy) and two due to missing data (in one patient, not all tests could be completed due to an eye condition, in another patient the MoCA could not be administered because it was accidentally missing in the test battery). The total sample therefore consisted of 95 HIV-1 infected patients. Table 1 shows relevant demographic characteristics and the scores on the MoCA and HDS. Mean age of the patients was 48.2 years. The mean nadir CD4 cell count was 213 cells/mm^3^ (IQR 100, 305) with all patients virologically suppressed on cART (<50 copies/mL). Using the Frascati criteria, 39 of the 95 patients (41.1 %) were classified as cognitively impaired. When the CIND criteria were applied, 22 of the 95 patients (23.2 %) were classified as cognitively impaired. The MoCA identified 33 of the 95 patients (34.7 %) as cognitively impaired, while the HDS identified 12 of 95 (12.6 %) patients with cognitive impairment, using the clinically established cutoff scores. Table 2 shows neuropsychological performance scores and impairments for the cognitive domains and all the tests in the neuropsychological assessment.

**Table 1:** Demographic variables and performance on the MoCA and HDS

Characteristic	HIV-infected patients (*N* = 95)
Age (years) [mean (SD)]	48.2 (10.1)
Sex	83 (87.4 %) male
	12 (12.6 %) female
Nadir CD4 cell count (cells/μL) [mean (IQR)]	213 (100–305)
Duration HIV infection (years) [mean (SD)]	9.83 (6.3)
Duration cART treatment (years) [mean (SD)]	8.44 (5.7)
Education level (median)^a^	6
Estimated IQ [mean (SD)]	98.2 (13.9)
Cognitive impairment cf. Frascati	39 (41.1 %)
Asymptomatic neurocognitive impairment (ANI)	34 (35.8 %)
Mild neurocognitive disorder (MND)	5 (5.3 %)
HIV-associated dementia (HAD)	0 (0 %)
Cognitive impairment cf. CIND	22 (23.2 %)
MoCA score [mean (SD)]	26.6 (2.3)
MoCA < cutoff	33 (34.7 %)
HDS score [mean (SD)]	13.8 (2.3)
HDS < cutoff	12 (12.6 %)

^a^Education level was recorded using seven categories that can be transferred to years of education: 1, 1–5 years; 2, 6 years; 3, 7–8 years; 4, 7–9 years; 5, 7–10 years; 6, 7–17 years; and 7, >18 years

**Table 2:** Neuropsychological performance: impairments on the cognitive domains and on each test per cognitive domain

Cognitive domains and tests	Mean (±SD)	*N* (%) impaired
Abstract reasoning	−0.38 (±1.03)	0 (0)
Raven Advanced Progressive Matrices	9.09 (±2.30)	0 (0)
Language	−0.05 (±1.02)	7 (7.4)
Letter Fluency Test (“K-O-M”)	40.13 (±13.20)	7 (7.4)
Speed of information processing	−0.11 (±0.80)	2 (2.1)
WAIS-III Digit-Symbol substitution	69.32 (±14.19)	10 (10.5)
TMT-A	30.54 (±9.98)	1 (1.1)
Stroop I and II	51.34 (±8.46)	1 (1.1)
Learning	−0.04 (±0.88)	5 (5.3)
RAVLT (total trials 1–5)	43.22 (±9.28)	10 (10.5)
LLT-R (total trials 1–5)	14.97 (±14.45)	2 (2.1)
Memory	0.00 (±0.77)	3 (3.2)
RAVLT (delayed recall)	8.69 (±3.02)	9 (9.5)
LLT-R (delayed recall)	0.64 (±1.68)	3 (3.2)
Executive functioning	−0.07 (±0.68)	0 (0)
Brixton	40.11 (±5.82)	1 (1.1
TMT-B	70.28 (±27.23)	4 (4.2)
BADS Zoo Map Test	11.38 (±4.10)	11 (11.6)
Stroop (interference)	0.61 (±0.33)	5 (5.3)
Attention/working memory	−0.09 (±0.71)	3 (3.2)
PASAT	32.44 (±9.39)	27 (28.4)
Corsi Block Tapping task (span forward and backward)	6.14 (±0.69)	0 (0)
WAIS-III Letter-Number Sequencing	11.12 (±3.20)	3 (3.2)
Motor	−0.09 (±0.89)	3 (3.2)
Pegboard ( dominant and nondominant)	79.26 (±12.48)	3 (3.2)
Visuoconstruction	−0.10 (±1.03)	7 (7.4)
Rey Complex Figure-copy	33.72 (±3.23)	7 (7.4)

Domain scores are presented as mean *z* scores ± SD; individual test scores are presented as mean raw scores ± SD. Cognitive domains were classified as impaired when a score of less than −1.65 SD was obtained in more than half of the tasks in that domain. Test scores were classified as impaired when a score of less than −1.65 SD was obtained on the age- and education-adjusted score

**Fig. 1:**
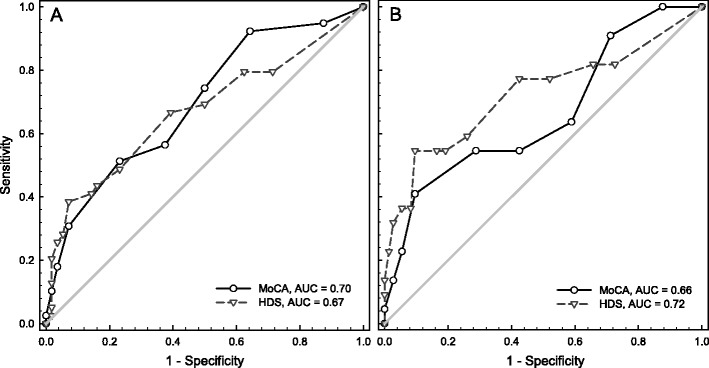
**a** ROC curves for the MoCA and HDS using several cutoff points in comparison with cognitive impairment classified with the Frascati criteria. **b** ROC curves for the MoCA and HDS using several cutoff points in comparison with cognitive impairment classified with the CIND criteria

Figure 1a, b shows the AUCs of the ROC analyses for the MoCA and the HDS in classifying cognitive impairment as measured with the extensive neuropsychological assessment, with impairment classified with both the Frascati and the CIND criteria in HIV-1-infected patients. All AUCs were statistically significant (Frascati: MoCA AUC = 0.70, CI = 0.59–0.80, *p* = 0.001; HDS AUC = 0.67, CI = 0.56–0.79, *p* = 0.005; CIND: MoCA AUC = 0.66, CI = 0.52–0.80, *p* = 0.024; HDS AUC = 0.72, CI = 0.58–0.86, *p* = 0.002). Different cutoff scores with the accompanying sensitivity and specificity for cognitive impairment are shown for both screening instruments and the two different criteria to classify cognitive impairment in Table 3. Cutoff scores with good sensitivity (>0.8) and an acceptable specificity (>0.6) could not be determined, however. The clinically established cutoff scores resulted in the following sensitivity (Se) and specificity (Sp) for the MoCA (<26) and HDS (<11) (Frascati: MoCA Se = 0.56, Sp = 0.63; HDS Se = 0.26, Sp = 0.96; CIND: MoCA Se = 0.55, Sp = 0.58; HDS Se = 0.36, Sp = 0.95). Using a cutoff of 27 for the MoCA slightly increased the sensitivity to 0.74 when the level of impairment was classified using the Frascati and to 0.64 when the CIND criteria were used. Also, the sensitivity of the HDS could be improved to 0.67 and 0.77 when a cutoff score of 14 was applied, for classification according to the Frascati and CIND criteria, respectively. The specificity of these cutoff scores remained low, however (0.41–0.61). Statistical comparison of the ROC curves for the two screening instruments using both the Frascati and the CIND criteria shows that the ROC curves did not differ significantly (Frascati: *z* = 0.025, CI = −0.10–0.15, *p* = 0.69; CIND: *z* = −0.064, CI = −0.22–0.10, *p* = 0.43).

**Table 3:** Cutoff points with different degrees of sensitivity and specificity for the MoCA and HDS in the identification of cognitive impairment using extensive neuropsychological assessment classified with the Frascati and CIND criteria

Cutoff	Sensitivity	Specificity
Frascati criteria		
MoCA		
25.5	0.51	0.77
26.5	0.56	0.63
27.5	0.74	0.50
28.5	0.92	0.36
HDS		
11.25	0.26	0.96
13.75	0.49	0.77
14.25	0.67	0.61
14.75	0.69	0.50
15.25	0.80	0.38
CIND criteria		
MoCA		
25.5	0.55	0.71
26.5	0.55	0.58
27.5	0.64	0.41
28.5	0.91	0.29
HDS		
11.25	0.36	0.95
13.75	0.59	0.74
14.25	0.77	0.58
14.75	0.77	0.48
15.25	0.82	0.34

## Discussion

This study examined the validity of the MoCA and HDS in relation to classification of cognitive impairment using both the Frascati and CIND criteria in a Dutch group of HIV-infected patients. With respect to discriminating patients with and without cognitive impairments classified using both the Frascati and the CIND criteria, the AUCs for both the MoCA and the HDS were statistically significant. Cutoff scores with both good sensitivity accompanied with a respectable specificity could not be determined, however. Furthermore, the AUCs for the MoCA and the HDS did not differ significantly regardless of the scoring criteria.

The proportion of HIV-infected individuals with cognitive impairments identified using the Frascati criteria (41.1 %) is similar to previous estimates of 33.3 and 52 % reported by Tozzi et al. (2003) and Heaton et al. (2010), respectively. When the more stringent CIND criteria are used, a much lower proportion of the patients was classified as being cognitively impaired (23.2 %). Using the current Frascati guidelines, the milder forms of HAND (MND and ANI) are defined by a performance 1 SD below the mean of normative scores in at least two domains. As mentioned previously, about 16 % of the normal population will by definition perform worse than 1 SD below the mean on a given test. In other words, around 16 % of a normal population will be classified as impaired when the Frascati criteria are used, which is an unacceptable false-positive rate (that is, 2–5 % is generally considered acceptable) (Lezak et al. 2012). Overestimation of the prevalence of HAND due to liberal classification criteria obscures the actual extent of cognitive deficits in HIV-infected individuals (Gisslén et al. 2011). Therefore, using more stringent criteria to determine cognitive impairment must be recommended. Alternative criteria to determine cognitive impairment in HIV-infected patients, first suggested in this study, are the CIND criteria. These criteria define impairment as an average performance >1 on the tasks in a cognitive domain. To obtain a score of >1 on a given domain, at least one of the tasks in that domain has to be severely impaired. The CIND criteria are therefore more stringent then the Frascati criteria. These might be used as an alternative to the Frascati criteria in classifying HIV-related neurocognitive impairment.

As noted previously, for both the MoCA and HDS, good sensitivity and specificity could not be found in distinguishing cognitively impaired from cognitively unimpaired patients. The sensitivity for both the MoCA and HDS was particularly low for the recommended cutoff scores when either the Frascati or CIND criteria were used to classify cognitive impairment. The sensitivity increased slightly when the thresholds for the MoCA and the HDS were raised, yet their specificity remained low, indicating a high risk of false-positive results. The current findings for the HDS are in line with previous studies who demonstrated poor prognostic values using the HDS to detect mild impairment in HIV-infected patients (Valcour et al. 2011; Bottigi et al. 2007; Zipursky et al. 2013). This screening tool was originally developed to detect HIV-associated dementia, and the performance characteristics to detect severe forms of cognitive impairment are modest to good (Berghuis et al. 1999). Morgan et al. (2008) showed that performance could be improved by adjusting for age and education, but the sensitivity remains modest (0.70) even after adjustment. To our knowledge, there are only a few studies that extensively investigated the validity of the MoCA in HIV-infected patients. Recent research of Overton et al. (2013) who also used extensive neuropsychological testing found somewhat higher sensitivity scores for the MoCA compared to the current study, but with comparable specificity levels. Another recent study by Milanini et al. (2014) found higher sensitivity and specificity levels than the current study, but investigated an older population of patients over 60 years. A study by Hasbun et al. (2012) showed moderate diagnostic accuracy for the MoCA (sensitivity 85 %, specificity 40 %). In contrast to the current study, the latter investigated ART-naive HIV-infected patients with high viral loads and high levels of comorbitities (hepatitis B and C), active drug use, depression, and unemployment. Koski et al. (2011) reported that the MoCA adequately measures cognitive ability as a global construct using Rasch analyses but showed poorer precision for measuring patients with higher cognitive ability. That is, half of the MoCA items were too easy for their high-functioning sample, resulting in ceiling performance. These items therefore contributed little to the measurement of overall cognitive ability in this group.

Regardless of the criteria used to classify the neuropsychological performance, milder forms of cognitive impairments continue to exist in a substantial amount of HIV-infected patients. Given the impact that these impairments may have on daily functioning and quality of life, the need for effective screening instruments to identify these patients remains high. Our study shows that simple tools developed for HIV-associated dementia are suboptimal in discriminating current HIV-infected populations. More comprehensive screening tools, like the MoCA, show mixed results in the literature. In our study, the validity of the MoCA was about similar to that of the HDS, but respectable sensitivity and specificity of this screening tool has been demonstrated for other patient groups. Sensitivity levels could be improved when the cutoff scores were increased, but the specificity levels remained low. A high sensitivity might be preferred in the clinical practice; however, combined with a low specificity, the risk of identifying patients without cognitive impairments as “impaired” is high. As a result, these tools are not recommended for use in the diagnostic process.

Our study was the first to investigate the validity of the MoCA in comparison with the HDS. Furthermore, unlike previous studies that have investigated the validity of MoCA and the HDS in HIV-infected patients, ours is the first that used both the Frascati and CIND criteria to classify cognitive impairment. Strengths of the study are the use of an extensive neuropsychological test battery in comparison to the short cognitive screening tools and the application of a symptom validity test. In order to obtain monetary compensation and/or service benefits, some patients might feign or exaggerate their neuropsychological deficits (Woods et al. 2003). Symptom validity tests are designed to be passable for all but the most severely impaired patients, given that the participant has provided adequate mental effort in the task. Also, this study had several limitations. Only HIV-infected patients who were on cART and were virologically suppressed for at least 1 year were included. These inclusion criteria were deliberately set to reduce the influence of other potentially confounding factors that may be present in uncontrolled HIV-infected patients, such as hepatitis B or C, syphilis, or malignancy. While our sample is representative for the majority of HIV-infected patients in the Netherlands (Van Sighem et al. 2013), to investigate detailed performance characteristics of cognitive screening instruments, a more heterogeneous group of patients with respect to severity of cognitive deficits might show better results. Secondly, although all patients were living independently at home and none fulfilled the criteria for dementia, we did not formally assess activities of daily living.

In sum, our study showed moderate sensitivity combined with poor specificity in detecting cognitive impairment with the MoCA and the HDS in HIV-infected patients, using both the widely applied Frascati criteria and the more stringent CIND criteria to classify cognitive impairment. On the basis of the present results, we cannot recommend these tools for the assessment of HIV-associated cognitive impairment.
